# Establishing foundations: Designing a long-term experiment to evaluate whether nestboxes assist population recovery of an endangered species after fire

**DOI:** 10.1371/journal.pone.0334130

**Published:** 2025-12-03

**Authors:** Jenna C.H. Ridley, Kara N. Youngentob, Karen Marsh, Maldwyn J. Evans, Tyrone Lavery, Kita Ashman, Ana Gracanin, David Lindenmayer

**Affiliations:** 1 Fenner School of Research and Society, Australian National University, Canberra, Australian Capital Territory, Australia; 2 Research School of Biology, Australian National University, Canberra, Australian Capital Territory, Australia; 3 School of BioSciences, The University of Melbourne, Melbourne, Victoria, Australia; 4 WWF-Australia, Melbourne, Victoria, Australia; National Museums of Kenya Ornithology Section, KENYA

## Abstract

The loss of hollow-bearing trees drives population declines of hollow-dependent species. Disturbances such as wildfire can exacerbate these declines. Artificial structures, like nestboxes, are a commonly used management tool that attempt to offset hollow loss. However, the effectiveness of nestboxes as a conservation strategy is rarely tested within an experimental framework. The endangered southern greater glider (*Petauroides volans)* is an obligate hollow-dependent arboreal marsupial that is highly sensitive to wildfire. Southern greater glider populations experienced high mortality and habitat degradation following the 2019/2020 megafires in south-eastern Australia. We established a long-term, landscape-scale experiment to test whether purpose-built nestboxes could assist the post-fire population recovery of the southern greater glider. We installed a total of 234 nestboxes at sites in East Gippsland (north-eastern Victoria) and Tallaganda (southern New South Wales). We matched nestbox sites to control sites. We undertook spotlighting surveys in both study areas and installed camera traps at a subset of nestboxes. We observed southern greater gliders using nestboxes in both regions, with substantially more observations in Tallaganda. These early results did not indicate a significant difference in the relative abundance of southern greater gliders between nestbox and control sites. In Tallaganda, we found more southern greater gliders in areas of lower fire severity. The longer-term outcomes of our study will inform the use of nestboxes as a tool to assist in the recovery of southern greater gliders following disturbance.

## 1. Introduction

Hollow-bearing trees play a critical role as an essential habitat element for cavity-dependent wildlife in many forest ecosystems [1–6]. Tree hollows are a valuable resource for fauna, providing shelter and safety from predators, which is critical to sustaining viable populations [3,5]. In Australia, hollows that serve these functions can take over 100 years to develop in eucalypt trees, and once lost, take significant time to replace [3,7–10]. Many Australian ecosystems have experienced declines in the abundance of tree hollows [11] stemming from major disturbances such as logging [12] and wildfire [13–15]. The loss of hollow-bearing trees at a landscape scale can lead to the localised extinction of hollow-dependent species [13,16,17].

Augmenting habitat with nestboxes may provide an alternative to the slow, natural development of tree hollows, and is a common management approach to assist hollow-dependent species [18–22]. Using nestboxes has proven to be successful for some threatened species conservation projects. For example, nestbox provisioning was identified as a key conservation action for the critically endangered Leadbeater’s possum (*Gymnobelideus leadbeateri*) [23]. Nestboxes have also been used by the endangered swift parrot (*Lathamus discolor*) [24].

Although nestboxes are frequently employed to support a range of threatened, hollow-dependent species [25], few studies have used rigorous and reliable experimental designs to accurately measure the long-term impacts of nestboxes on a species population [20,26]. There have also been several nestbox projects with unexpected results. For example, a study of Victoria’s mountain ash (*Eucalyptus regnans*) forests, found the majority of nestboxes (68.8%) were not used or occupied by any arboreal marsupials [27]. Moreover, limitations and unintended outcomes from nestbox provision can occur, such as uptake by non-target species [27,28] including pest animals [29], thermal stress [30], mortality [20,31], and lack of use by the targeted species [32].

The endangered southern greater glider (*Petauroides volans*) is a hollow-dependent, nocturnal, arboreal marsupial that occupies eucalypt-dominated forests in eastern Australia [33–35]. Population declines of this species have been observed across their range [36,37], including following the 2019/2020 megafires. This major fire event burnt ~8.9 million hectares of native forest [38,39], further reducing southern greater glider populations, prompting their uplisting nationally from Vulnerable to Endangered in 2022 [40]. Fire can directly reduce hollow abundance through hollow-bearing trees being consumed or falling [7,41], but conversely, fire can be a hollow-forming process [42]. In areas burnt by moderate or high severity fire, losses of hollow-bearing trees may be especially pronounced compared to areas affected by low severity fires [41,43,44]. This loss may be exacerbated by salvage logging, a practice which involves removing trees after natural disturbance for economic benefit [45,46]. Post fire, there was concern about the persistence of the southern greater gliders [47], and the loss of critical habitat features (i.e., tree hollows) [48].

Nestboxes have been recommended as a potential conservation action for the southern greater glider following the loss of tree hollows [22,48,49]. Here, we describe the establishment of a long-term experiment to assess the efficacy of nestbox installation as a tool to support the recovery of southern greater glider populations in recently burnt habitat.

Using a combination of baseline data and survey data collected post nestbox deployment, we asked the following questions:

1. Do southern greater gliders use purpose-built nestboxes?
2. Did non-target species use nestboxes purpose-built for southern greater gliders?
3. Did the relative abundance of southern greater gliders increase in treatment sites (i.e., with nestboxes) compared to control sites (i.e., without nestboxes)?
4. Did fire severity affect the relative abundance of southern greater gliders in the study area?

At the outset of this study, we predicted that southern greater gliders would use purpose-built nestboxes based on their successful use of a similarly designed prototype deployed in unburnt East Gippsland forests [50]. We postulated that a modified design with a smaller entrance size, would exclude larger competitors, such as the common brushtail possum (*Trichosurus vulpecula*) or mountain brushtail possum (*Trichosurus cunninghami*) [25,51]. We expected a negative relationship between high fire severity and the relative abundance of southern greater gliders, based on the findings of other post-fire studies [43,52]. Given that southern greater gliders have high site fidelity, relatively small home ranges, and are slow to reproduce [49,53,54], we did not expect to document an increase in southern greater gliders in areas with nestboxes in the short time span of our study. Nevertheless, we provide critical baseline data for ongoing monitoring of long-term outcomes.

## 2. Materials and methods

This research was approved by the ANU Animal Ethics Committee (protocol number A2021/15). Access agreements were granted by the Department of Environment, Land, Water and Planning (DEECA), Parks Victoria, NSW National Parks, and the Forestry Corporation of NSW.

### 2.1. Study region

**Fig 1:**
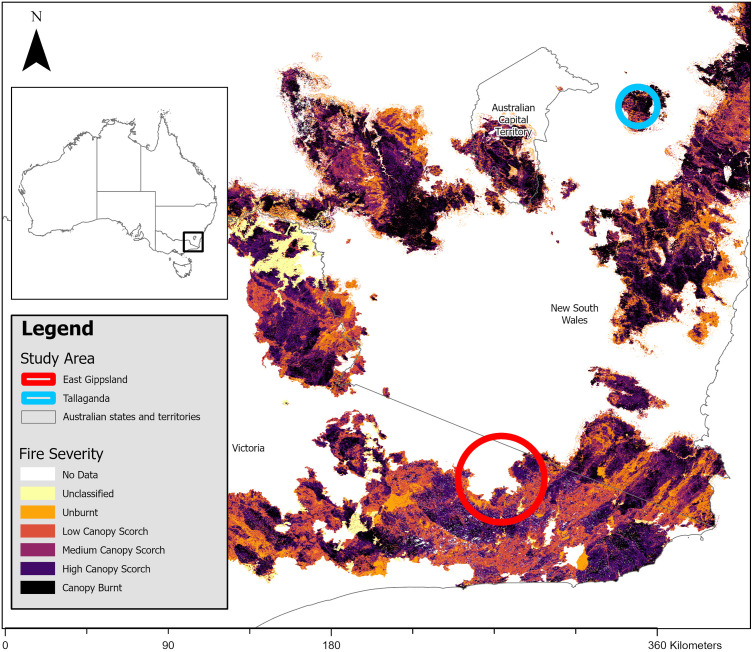
East Gippsland (red) and Tallaganda (blue) study areas, with the 2019/2020 megafire scar displaying the discrete fire severity classes [55,56]. The inset map of Australia has a square around the highlighted study region in the main map.

We conducted this study in eucalypt-dominated forests in two study areas affected by the 2019/2020 megafires (Fig 1). We established one study area in the East Gippsland Shire (hereafter called East Gippsland) in Victoria, located on the lands of the Bidwell, Yuin, Gunaikurnai, and Monero (Ngarigo) First Nations people. Our study was in a small part of the wider East Gippsland region around the Errinundra Plateau in the East Gippsland Uplands Bioregion [57], near the town of Bendoc (37.1468°S, 148.8816°), approximately 10 km south of the New South Wales (NSW) border (Fig 1). The dominant forest types include shrubby dry, damp, and wet forest and the area receives an average rainfall of 800–1200 mm, increasing to 1400 mm in some of the areas around the Errinundra plateau [57,58]. Key overstorey species commonly include a mix of stringybark (*E.globoidia –* *White Stringybark, E. macrorhyncha –* *Red Stringybark, E. obliqua –* *Messmate*), gum (*E. denticulata –* *Errinundra Shining Gum, E.dives –* *Broad-leaved Peppermint, E.rubida –* *Candlebark*), and ash-type (*E. regnans –* *Mountain Ash*, *E. sieberi* – Silvertop Ash) eucalypt species with a broad-leaved shrubby understorey mix (*Cassinia* sp., *Coprosmo* sp. – Mirror Bush, *Leucopogon* sp. – Beard Heath, *Tasmannia* sp. - Pepperbush), particularly in the wetter areas [57]. The elevational range of the study area was from approximately 600 m up to 1400 m [57]. The East Gippsland population of southern greater gliders has declined substantially in recent years and persists at relatively low densities in some areas [59].

Our second study area was at Tallaganda National Park and State Conservation Area in NSW (hereafter Tallaganda) on the lands of the Ngarigo and Walbanga First Nations people, located 30 km southeast of Canberra (35.2802° S, 149.1310° E) (Fig 1) [60]. Tallaganda is on the Great Dividing Range of eastern Australia and supports a range of vegetation types, including moist and dry sclerophyll forest [60]. Dominant overstorey species include *E. fastigata* (Brown Barrel), *E. radiata* (Narrow-leaved peppermint) and *E. viminalis* (Ribbon gum), with an understorey dominated by *Acacia* spp. [60]. The elevational range in Tallaganda is 700 m to 1400 m [60] and the average annual rainfall is typically ~900 mm [61]. Detection rates of southern greater gliders in Tallaganda are typically high [62], and population density is also considered to be high [60].

### 2.2. Pre-site selection surveys

To guide site selection, we compiled historical locations of southern greater gliders [63–68], and post-fire southern greater glider observations [69]. We then confirmed locations where southern greater gliders persisted post megafire, using spotlighting surveys throughout East Gippsland [59](S1 Table in S1 File) and Tallaganda (S2 Table in S1 File). Surveys began one hour after sunset, in which we identified all arboreal marsupials (S3 Table in S1 File) and recorded the distance and compass bearing to each individual. Where southern greater gliders were confirmed as present, we selected our individual study sites within 1.5 km of these sightings, according to the additional stratified criteria outlined below.

### 2.3. Study sites

**Fig 2:**
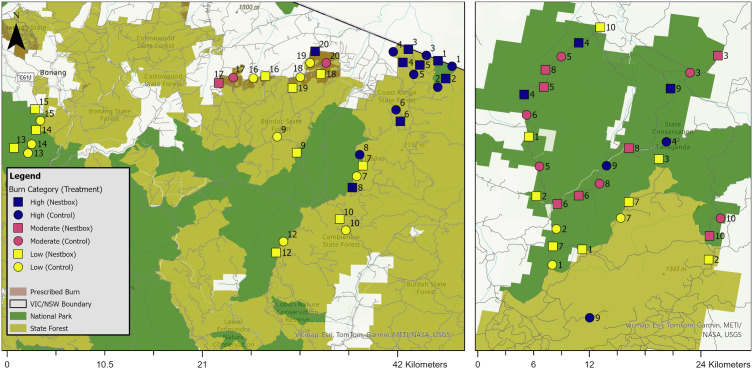
Nestbox (square) and control (circle) sites across (Left) East Gippsland and (Right) Tallaganda. We used burn percentage to assign sites to one of three fire severity classes: low (<35%; yellow), moderate (36 to 70%; pink) and high (>71%; purple). The numbers represent the matched pairs or triplets.

In East Gippsland, we selected 40 sites, consisting of 20 matched pairs. We matched one treatment site (i.e., with nestboxes; n = 20 sites) to one control site (i.e., without nestboxes, n = 20 sites) based on site and environmental attributes (Fig 2, S4 Table in S1 File). Due to insufficient southern greater glider observations in areas burnt in the 2019/2020 megafires, we located some sites within a 2019 or 2021 prescribed burn scar. In Tallaganda, we selected 30 sites within the 2019/2020 megafire burn extent (Fig 2, S5 Table in S1 File). Due to the comparatively smaller extent of the burn area in Tallaganda, we had a 2:1 ratio, with two nestbox sites matched to one control site. All sites in both study areas were >1 km apart to reduce the likelihood of detecting the same southern greater glider across multiple sites.

We paired sites based on the following spatial layers, Ecological Vegetation Class (EVC) [70,71], fire severity [55,56], forest age [72], and field assessments. We selected sites in areas that were not part of logging plans in the next five years and had not been logged for at least 30 years. We created matched pairs of sites and then, using a random number generator, allocated one site in each pair as a treatment site and the other as a control site.

We established 300 m long, off-track transects with flagging tape and reflective markers at each site. To minimise edge effects and road disturbance, we positioned the start of each site more than 25 m from a road or track. In some areas, we had to establish the transect parallel to a road. Where this occurred, we positioned all transects and nestboxes at a minimum of 50 m from the road/track.

We collected environmental data at each site. This included measuring the percentage of the site that was burnt (percentage burnt) and whether canopy foliage was present or absent (canopy burnt).

### 2.4. Spotlight surveys

In East Gippsland, we surveyed all treatment and control sites prior to nestbox deployment (04/01/22 to 27/03/2022; 40 sites). In Tallaganda, we surveyed 10 of the 20 nestbox sites prior to, or within two days of, nestbox deployment. However, due to Covid-19 travel restrictions and weather constraints, we were unable to survey 10 nestbox sites prior to nestbox installation.

Our spotlighting surveys were consistent with the double-observer survey method as outlined in a previous study (see [73] for full methodology). We walked at a pace of approximately 10 min/100 m (i.e., 30 minutes for 300 m), and the second observer began surveying a transect 15 minutes after the first observer had started. To ensure animals were not double counted, we recorded the colour morph, position in the tree, the observer’s location (using Avenza maps, [74]) and the bearing and distance to the animal from the observer’s location. We recorded the bearing using a compass and the distance using a laser range finder. Immediately after each survey, both observers used this information to identify each southern greater glider along the transect and ensure that individuals were not double counted. However, this was more challenging to implement in Tallaganda than in East Gippsland due to the high-density of ‘black and white’ morph individuals on a single transect. Due to inconsistencies between survey rounds, including how data were collected between different observers, and the independent detection challenges, we elected not to analyse the double observer data using distance sampling in this study (Refer to 2.7 Survey Indices for a description of the metric we used for greater glider counts).

### 2.5. Nestbox deployment

We installed purpose-built nestboxes targeted for southern greater gliders [50]. Southern greater gliders are particularly sensitive to overheating [75–77]. Nestboxes have been shown to be hotter than natural hollows [22] and may contribute to heat stress in animals [78]. Therefore, the nestbox design we utilised was designed and tested prior to deployment to prevent adverse effects, such as overheating [50]. The nestbox design also incorporated a relatively small entrance size suitable for entry by southern greater gliders but which precluded access by larger species of arboreal marsupials [25,51]. The entrance size also was large enough to be potentially less appealing to very small arboreal marsupials [18].

We installed six nestboxes at each treatment site; two nestboxes each within 50 m of the 50 m,150 m and 250 m points along our transects (S1 Fig in S1 File). We deployed one nestbox per tree at a height > 8m on the south-eastern aspect of a mature eucalypt tree [79,80] which typically had a trunk width exceeding the width of the nestbox. We deployed 120 nest boxes (6 per transect; 20 transects) in Tallaganda between the 21^st^ of March and the 2^nd^ of April 2022 and 114 nestboxes (6 per transect; 19 transects) in East Gippsland between the 28^th^ of March 2022 and 12^th^ of May 2022.

### 2.6. Post-deployment surveys

We conducted spotlighting surveys on the transects in East Gippsland almost one-year post-nestbox deployment using the same methodology as pre-deployment surveys (Round 2;16/01/2023 to 6/02/2023; 36 sites). During nestbox deployment, we removed two sites because of accessibility constraints, and we were unable to access an additional two sites in the post-deployment survey. This left 36 sites in East Gippsland for analysis (18 nestbox sites, 18 control sites). We completed spotlight surveys at the Tallaganda sites in late October/early November of 2022, approximately 6–7 months post-nestbox deployment. We surveyed all 30 sites using the same methodology as pre-deployment surveys and used all 30 sites in the analysis.

### 2.7. Survey indices

From our field surveys, we calculated the highest count of independent southern greater gliders seen per survey, using the most southern greater gliders seen by either observer per survey. We did not count dependent young (in pouch or on back) as independent from their mother. We used raw spotlight counts as an index of relative abundance (see [73]), recognising that this does not represent true abundance but allows valid comparisons between treatments and sites when survey effort and detectability are consistent.

### 2.8. Camera trapping

We installed 54 Reconyx IR cameras (HP2X Professional) with a 900–1200 mm focus on a subset of sites in East Gippsland (14 sites, 28 cameras) and Tallaganda (13 sites, 26 cameras). At each site, we installed one camera on one nestbox at the 50m point along a transect and one camera on one nestbox at the 250 m point along a transect. Previous work indicates that southern greater gliders might experience increased competition from larger species of arboreal marsupials at habitat edges, for example, along cleared roads or tracks [81]. Therefore, our placement of cameras might allow us to investigate this potential competition issue in the future. We set cameras to ‘night only’ to conserve battery life on the trigger setting, with two pictures per trigger and no delay. We installed cameras during the initial nestbox installation in East Gippsland, and between 12^th^ to the 16^th^ of July 2022 in Tallaganda, approximately three to four months after nestbox installation due to the previously mentioned logistical constraints.

### 2.9. Image coding

To sort and code our images, we used Reconyx Map view Professional [82] and Timelapse [83]. We discarded any blank images (no animal). We sorted images into ‘mammal’ or ‘no mammal’ and discarded those with no mammal present. We coded each individual image with species, behaviour (on tree, on nestbox, looking at/in nestbox, inside nestbox, entering or exiting nestbox), colour morph (if southern greater glider), age (adult/juvenile/juvenile on back of adult). We then sub-categorised the behaviour images into how the nestbox was being used: “use nestbox” (in, entering, exiting), “interact with nestbox” (looking at/in, on nestbox) and “no interaction with nestbox” (on tree, jumping/gliding). We used one image as a single detection but used presence per day as our main index of use. We counted only one southern greater glider per day unless there were multiple individuals in the one image, or there were characteristics that enabled differentiation between individuals, e.g., different colour morphs. Upon reviewing our data, we found that seven cameras in East Gippsland were set up with half the nestbox entrance outside the field of view. We chose to include these data in our analysis, however, the set up may have impacted identification of some species, as less of the animal was visible. In Tallaganda, there were six cameras where the SD card filled prior to the retrieval date and this likely impacted the amount of data collected.

### 2.10. Statistical analysis

#### 2.10.1. Camera monitoring [Question 1 and 2].

We used the CamtrapR program [84] and the packages dplyr [85] and ggplot2 [86] to create summary statistics of the camera trapping data in R [87]. We investigated the occupancy of nestboxes by target and non-target species. We were unable to gather data on the time period between nestbox installation and use by southern greater gliders at Tallaganda given the ~ 3-month delay in camera deployment at that location. We calculated the number of trap nights and filtered the image datasets based on the start and end dates, removing any problem periods. Problem periods included times where the camera had stopped taking images or had been moved significantly from the original field of view. For each species, we calculated the relative frequency of detections (number of images/total trap nights), and the percentage of trap nights detections were recorded at each study site.

#### 2.10.2. Spotlight surveys [Question 3 and 4].

To test for the effects of nestbox installation, and fire severity on the relative abundance of southern greater gliders, we fitted generalized linear mixed models, assuming Poisson error distributions, implemented in the glmmTMB package [88] in R. We used *highest count* of southern greater gliders as the response variable and analysed Tallaganda and East Gippsland data separately. We analysed the two sites separately because the nestbox–control designs differed (1:2 matching in Tallaganda vs. 1:1 in East Gippsland) and because the populations differed in density and context. Pooling would risk pseudoreplication and with only two sites, ‘site’ could not be modelled as a random effect, meaning that separate analyses more accurately reflect the independence of the two experiments. We used *treatment* (nestboxes or control) to define the category of each site and *round* (pre or post) to define whether the survey was completed prior to (pre; round 1) or after (post; round 2) nestbox deployment. We also tested the effect of *burn percentage* (%), a continuous variable recorded in the field to represent the percentage burnt of each site. We included both *site ID* and *rep* (the matched sites) as random intercept factors to allow for dependence between repeated surveys at the same site and between matched sites. We assessed model performance by inspecting the histograms of residuals and checking for overdispersion and zero inflation using the ‘performance’ package in R [89].

For Tallaganda, we removed twelve surveys from the analysis where nestbox deployment occurred prior to spotlighting. This was to ensure consistency in the analysis ensuring that spotlighting seasons before and after deployment were matched.

## 3. Results

### 3.1. Pre-Site selection surveys

We encountered southern greater gliders more frequently in Tallaganda than in East Gippsland. Overall, we detected 34 individual southern greater gliders in ~50 km of surveys in East Gippsland (S1 Table in S1 File) and 84 individual southern greater gliders in ~ 8 km in Tallaganda (S2 Table in S1 File). Despite there being many historical records of southern greater gliders in East Gippsland, we found very few animals in these areas.

### 3.2. Camera trapping at nestboxes

#### 3.2.1 Question 1: Did southern greater gliders use purpose-built nestboxes?.

**East Gippsland.** In East Gippsland, we deployed cameras for a total of 13,739 trap nights. The Feathertail glider (*Acrobates frontalis or Acrobates pygmaeus*) was the most frequently detected species (100% of sites; S6 Table in S1 File), followed by the mountain brushtail possum (*Trichosurus cunninghami*), southern greater glider, and inland sugar glider (*Petaurus notatus*) (Table 1). Overall, we detected six species of arboreal marsupials, plus *Antechinus* spp. and bats, which we did not identify to species level. Interestingly, *Antechinus* spp. were detected only on camera, whereas the common ringtail possum was detected only during spotlighting surveys (S6 Table in S1 File). Detection of our target species was similar between survey methods. However, per site, spotlighting yielded better detection rates in East Gippsland (S6 Table in S1 File).

**Table 1:** The relative frequency (number of images/total trap nights) and percentage of trap nights each fauna species was detected at each study site during camera trapping.

		East Gippsland		Tallaganda	
Scientific Name	Common Name	Relative Frequency	Trap nights detected (%)	Relative Frequency	Trap nights detected (%)
*Acrobates* spp.	Feathertail Glider	**0.489**	**10.39**	0.032	1.49
*Antechinus* spp.	*Antechinus*	0.003	0.12	0.002	0.04
*Petauroides volans*	Southern Greater Glider	0.109	0.72	**3.031**	**22.85**
*Petaurus australis*	Yellow-bellied Glider	0.002	0.08	0	0
*Petaurus notatus*	Inland Sugar Glider	0.109	1.66	0.009	0.34
*Trichosurus vulpecula*	Common Brushtail Possum	0	0	0.002	0.04
*Trichosurus cunninghami*	Mountain Brushtail Possum	0.12	0.37	0	0
Unknown	Bat spp.	0.008	0.22	0	0

**Fig 3:**
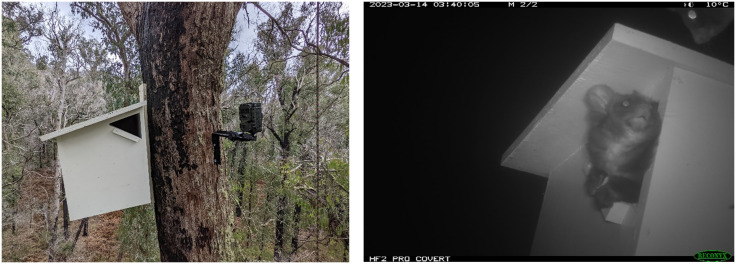
(Left) Camera installed facing a nestbox in East Gippsland (Photo Credit: M Cobden), (Right) Southern greater glider inside purpose-built nestbox, while another greater glider looks toward it from the tree.

Cameras detected southern greater gliders at nine of 28 individual nestboxes (32%), in five of 14 sites in East Gippsland. The shortest period for detecting a southern greater glider at a nestbox after installation was 10 days. We did not observe definitive use of the nestbox (i.e., entering, exiting or inside) until 307 days after installation (Fig 3; S2 Fig in S1 File). Only one camera detected nestbox occupancy (entering/exiting/inside) by a southern greater glider (S2 Fig in S1 File). This nestbox was occupied by the inland sugar glider prior to the southern greater glider observation (S2 Fig in S1 File). Two of the nine cameras at nestboxes (22%) with southern greater gliders did not have the entrance in full view, which may have resulted in some aspects of behaviour or nestbox occupancy being missed.

**Tallaganda.** In Tallaganda, we deployed cameras at nestboxes for a total of 2,350 trap nights. During this time, some cameras collected data for a short period (<30 days, n = 5). Five cameras did not detect any arboreal marsupials. We observed repeated use of nestboxes by southern greater gliders, with the relative frequency of detections being 3.03, far higher than any other species (Table 1). In Tallaganda, southern greater gliders were the most common species detected by cameras and spotlighting surveys (S6 Table in S1 File). Initial observations of nestbox occupancy were during camera deployment in July 2022 (~3 months after nestbox deployment). Cameras detected southern greater gliders at 16 of 26 nestboxes (61.5%), which was 12 of 13 sites. We confirmed that southern greater gliders were using (entering/exiting/inside) nine of the 26 monitored nestboxes across the study period of ~six months (37.5%; S3 Fig in S1 File).

**Fig 4:**
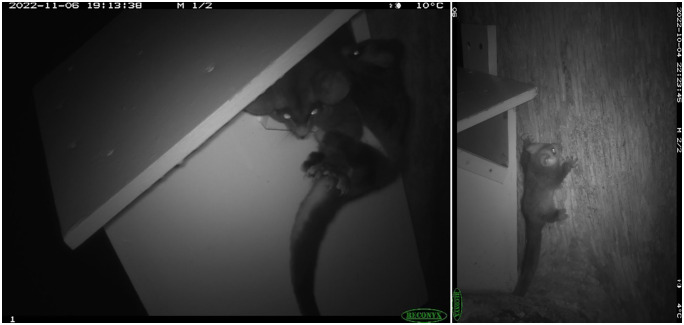
Southern greater glider female adult and juvenile at nestbox sites in Tallaganda. (a) Mother and juvenile, and (b) juvenile climbing the tree outside of the nestbox.

We observed juvenile southern greater gliders on camera at two nestboxes (Fig 4). They appeared to be on the back of the mother and spent time moving in and out of the nestbox. At one site (03B), a juvenile was detected on camera for 31 days out of a 47-day period (From 25/09/2022 to 11/11/2022), and at the second site (07A) was detected on 14 days out of a 34-day period (From 31/10/2022 to 3/12/2022). We occasionally observed two similar sized southern greater gliders entering and exiting nestboxes. In August-September and October-November 2022, we detected two adults sharing a nestbox on several occasions at two different locations.

#### 3.2.2. Question 2: Did other species occupy purpose-built nestboxes?

**Fig 5:**
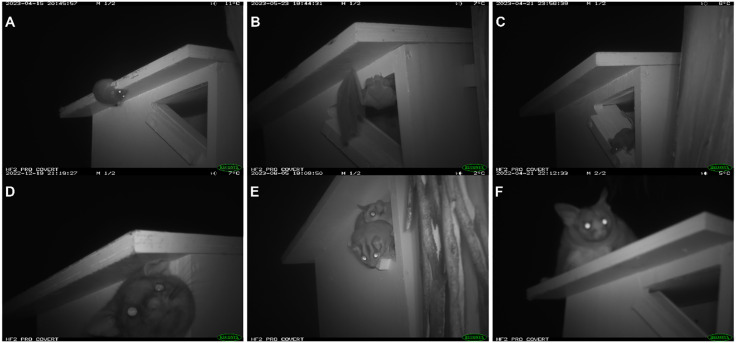
Mammal species detected at nestboxes in East Gippsland. A) Antechinus spp. inspecting nestbox, B) Bat spp.in a nestbox entrance, C) Feathertail glider in a nestbox entrance, D) Mountain Brushtail Possum exiting nestbox, E) Inland sugar glider and dependent young inside a nestbox, F) Yellow-bellied glider on top of nestbox.

**East Gippsland.** In East Gippsland, inland sugar gliders occupied eight nestboxes, and a mountain brushtail possum occupied one nestbox (Fig 5). We observed inland sugar gliders using two of the eight nestboxes for a 21 day (23/10/2022 to 21/03 2023) and a 46-day period (30/11/2022 to 09/08/2023) (Fig A2). Both nestboxes were on the same transect (Site 17NB; Fig A2). We observed inland sugar gliders with their offspring in nestboxes (Fig 5) and bringing vegetation into the nestboxes. We also detected a mountain brushtail possum using a nestbox (06NB_250m) from 19/12/2022 to 12/09/2023. We observed several bats on camera, although it is unknown if they used nestboxes to roost.

**Tallaganda.** In Tallaganda, southern greater gliders were the most observed species using the nestboxes (Table 2). In both Tallaganda and East Gippsland (Fig 5), we interpreted feathertail gliders as ‘using’ the nestbox, after observing them going in and out of different nestboxes. This species’ movements were more rapid than those of southern greater gliders and they appeared to exit soon after they entered. We assumed, therefore, that they were not occupying these nestboxes.

**Table 2:** Generalised linear mixed model results for East Gippsland and Tallaganda using highest count as the response variable.

	East Gippsland				Tallaganda			
	*Estimate*	*Std. error*	*z value*	*P-value*	*Estimate*	*Std. error*	*z value*	*P-value*
(Intercept)	-0.871	0.65	-1.341	0.18	1.577	0.29	5.438	5.4e-08 ***
Treatment	-1.422	0.773	-1.841	0.066	0.322	0.256	1.256	0.2091
Survey round	-0.406	0.456	-0.888	0.37	-0.357	0.285	-1.254	0.2100
Burn Severity	-0.001	0.009	-0.076	0.94	-0.011	0.004	-2.698	0.007**
Treatment: Round (Pre)	0.406	0.935	0.434	0.665	0.073	0.383	0.191	0.849

The estimate, standard error, z value and p value are shown for each variable in each model. The Tallaganda model was zero-inflated, with the ratio of observed zeros a 3, and predicted zeros a 4 (Ratio 1.33) [89]. Significant values are shown by an * and in bold.

### 3.3. Spotlighting surveys

**East Gippsland.** We observed a total of 29 southern greater gliders across both spotlighting survey rounds in East Gippsland (i.e., 36 sites, surveyed two times each). We detected southern greater gliders in 27.8% of pre-nestbox deployment surveys and 22.2% of post-nestbox deployment surveys. The highest number of southern greater gliders detected at one survey site was six (site 16C).

**Tallaganda.** We detected 245 southern greater gliders across 60 surveys (i.e., 30 sites surveyed twice each). We detected southern greater gliders in 90% of pre-deployment surveys and 93.3% of post-deployment surveys; the highest number detected was 14 (1 survey during post-deployment surveys; site 02B).

#### 3.3.1. Question 3: Did relative abundance of southern greater gliders increase in treatment sites (i.e., with nestboxes) compared to control sites (i.e., without nestboxes) post treatment?.

There was no significant interactive effect of treatment or control on the relative abundance of southern greater gliders in East Gippsland or Tallaganda (Table 2). Mean relative abundance of southern greater gliders was not predicted to change at nestbox sites post treatment in either East Gippsland or Tallaganda (S4 Fig in S1 File). This non-significant result indicates that there was no evidence for an increase in southern greater glider relative abundance in treatment sites post deployment during this time (Table 2; S4 Fig in S1 File).

#### 3.3.2. Question 4: Does fire severity affect the relative abundance of southern greater gliders in the study area?.

**Fig 6:**
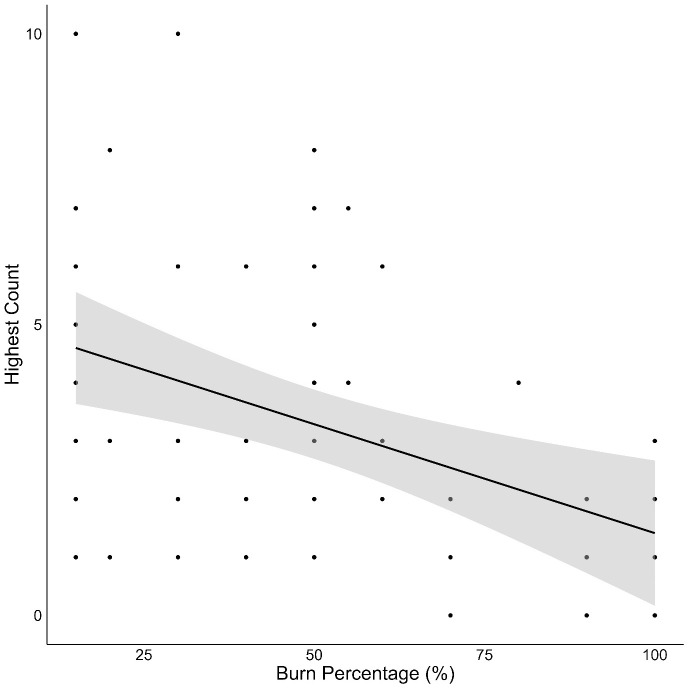
Relative abundance of the southern greater glider in relation to burn percentage (%) at survey transects in Tallaganda using both pre- and post-treatment survey data. Points represent the number of southern greater gliders detected at different burn percentages. The black line is the fitted response and 95% confidence intervals from a generalised linear mixed model.

We found no significant effect of burn percentage in East Gippsland. However, in Tallaganda there were significantly fewer southern greater gliders in areas with higher burn percentage than those with a lower burn percentage (Table 2; Fig 6).

Rates of southern greater glider detection were markedly different between our two study populations, with the species being detected much more frequently in Tallaganda than in East Gippsland. In both study areas, southern greater gliders occupied our purpose-built nestboxes. We found no significant difference in the numbers of southern greater gliders between treatment and control sites prior to nestbox (treatment) installation. We did not find an increase in southern greater glider detections in areas with nestboxes compared to those without, possibly due to the short duration of our study (≤ 12 months) relative to the slow reproductive and recruitment rates of greater gliders [53].

### 4.1. Nestbox use by southern greater gliders

We observed higher nestbox use (i.e., definitive occupation) by southern greater gliders in Tallaganda, where southern greater gliders were more frequently detected, compared to East Gippsland. However, we observed southern greater gliders investigating several nestboxes at sites in both regions, which may indicate occupation that was not captured by the cameras. We used cameras to monitor only a subset of nestboxes, and therefore our observations of occupation are limited to those nestboxes.

Differences in hollow abundance or availability may explain the differences in nestbox uptake between study areas. Tree hollows, which provide denning and nesting resources for several arboreal marsupials are fundamental to the survival of southern greater gliders [8,90]. [44] found that hollow occurrence and abundance decreased in areas burnt at high severity in East Gippsland. Whilst a similar amount of area was burnt at high severity in Tallaganda, the abundance of hollows has not been quantified. More detailed and extensive hollow counts at all sites will aid future work in determining whether hollow abundance is a driver of these differences in uptake. Alternatively, hollow quality could be an influential factor and may be important to investigate in future studies. A nestbox study conducted on a private property connected to Tallaganda National Park found 100% occupancy of 30 nestboxes within a year [22]. This area had been affected by clearfell logging and high severity fire, however, the authors proposed the abundance of quality hollows was low [22].

The relative abundance of southern greater gliders in Tallaganda potentially increased the likelihood of nest box use. This may relate to the availability of hollows, or other factors, such as variations in local climate and the nutritional quality of landscapes [76,91,92]. It is possible that these other factors have contributed to higher population densities of southern greater gliders in Tallaganda compared to East Gippsland. A higher population density may create more competition for hollows and result in higher nestbox uptake by the species.

We documented adult (similar sized) southern greater gliders sharing nestboxes, as reported in earlier studies [53,93,94]. The social organisation of arboreal marsupials can be assigned to three broad types; colonial, solitary, or pairs [95]. Southern greater gliders have primarily been recorded as a solitary species, although they may be more social than previously recognized. Early studies have suggested that arboreal marsupials alter their social organisation depending upon resource availability [95]. It is also possible that the two similar sized individuals were a parent and a previous years’ offspring, or a mated pair [96], indicating breeding may have taken place within nestboxes. At two nestboxes in Tallaganda, we observed southern greater gliders with dependent young (Fig 4). Female southern greater gliders may be more likely to utilise nestboxes during the breeding season. This result has been supported by a similar study where high rates of juveniles were recorded in nestboxes during spring and summer [22]. Understanding these behaviours and how they affect population dynamics should be an important consideration for future studies of the species, and how nestboxes can be implemented successfully.

### 4.2. Nestbox use by non-target species

Both of our study areas are home to diverse assemblages of arboreal marsupials (S3 Table), most of which are hollow dependent and may compete with the southern greater glider for this essential resource. We observed occupancy by non-target species primarily at nestboxes in East Gippsland. Notably, we did not observe occupancy of nestboxes by non-target species in Tallaganda. This could be a result of the differences in numbers of southern greater gliders between the two populations. The population at Tallaganda was substantially higher than in East Gippsland, and southern greater gliders were the most common species observed (Table 2). This could have resulted in southern greater gliders quickly occupying the nestboxes in Tallaganda and outcompeting other species for residence. A recent study found southern greater gliders to rapidly colonise nestboxes within an average of 34 days after nestbox deployment [22].

Inland sugar gliders were the most common nestbox inhabitants in East Gippsland, occupying approximately 29% of monitored nestboxes. However, compared to other studies where non-target species are the most observed occupants [97], we observed low levels of non-target species uptake. The low rate of non-target species uptake may be attributed to the design of our nestboxes, which were constructed specifically for the southern greater glider. We built nestboxes with a small entrance to exclude larger competitors such as brushtail possums; however, this design did not exclude smaller species such as inland sugar gliders [18]. Ongoing camera monitoring will assist in determining whether the use of nestboxes by non-target species changes over time.

### 4.3. Nestboxes supporting recovery

We did not find an increase in southern greater gliders numbers in areas with nestboxes compared to those without during the first round of post-treatment monitoring (Table 3.2). This was expected due to the low fecundity [53], high site fidelity, and small home ranges [49] of the southern greater glider. A prolonged period of monitoring will be needed to determine if the deployment of nestboxes leads to an increase in abundance in the medium to long term.

### 4.4. Fire severity and glider abundance

We found southern greater gliders in sites ranging from low to high burn percentages in both study areas. This is a promising indication that nestboxes may support recovery of fire-sensitive species, particularly with projected increases in the occurrence and severity of wildfires [98]. However, we did find that fire severity influenced southern greater glider occurrence in Tallaganda, with fewer animals in areas with a higher burn percentage (Fig 6). This result is consistent with other studies of southern greater gliders [37,43,44,52]. We did not find the same relationship between burn percentage and southern greater glider numbers in East Gippsland. This may be attributed to the fewer records of southern greater gliders and a reduced number of sites supporting the species, making it difficult to obtain a statistically robust answer. The direct impact of fire leading to mortality of individual southern greater gliders is challenging to mitigate, therefore, proactive measures to reduce the risk of increased fire severity in future events are important [99].

## 5. Conclusion

Our study highlights the potential for nestboxes to be a successful management tool to supplement natural hollows after disturbance. It also demonstrates that different populations of the same species may respond to the implementation of nestboxes differently, likely due to environmental factors, such as hollow availability. Long-term monitoring at these sites over the next decade will be imperative to assess southern greater glider population changes in response to nestboxes and confirm whether nestboxes are an effective management tool for this endangered species longer term.

## Supporting information

S1 File Supporting Materials: Supporting information file including six tables (S1 Table – S6 Table) [100,101] and four figures (S1 Fig – S4 Fig). (DOCX)
