# Supplementary material for: Establishing foundations: Designing a long-term experiment to evaluate whether nestboxes assist population recovery of an endangered species after fire
Source: PLoS One. 2025 Dec 3;20(12):e0334130. doi: 10.1371/journal.pone.0334130 (PMC12674562; doi:10.1371/journal.pone.0334130)
Supplement: S1 File — (DOCX) [file pone.0334130.s001.docx]

# Supporting Materials

##### **S1 Table: The number of spotlighting surveys conducted in East Gippsland between April and December 2021, the transect length, and number of individual southern greater gliders detected. Survey method and number of observers per survey differed. Surveys were conducted on foot (except for those marked with * which were driven) along tracks and roads. Some surveys were on foot through the forest. These surveys were conducted within the Bendoc, Bonang, Goongerah and West Cann area of East Gippsland, Victoria.**

| Transect Length (m) | Number of Surveys | Number of Individuals |
| --- | --- | --- |
| 120 | 1 | 0 |
| 200 | 46 | 8 |
| 220 | 1 | 0 |
| 270 | 1 | 1 |
| 300 | 15 | 4 |
| 350 | 1 | 0 |
| 380 | 1 | 0 |
| 400 | 1 | 0 |
| 450 | 1 | 0 |
| 470 | 1 | 3 |
| 500 | 38 | 3 |
| 550 | 3 | 3 |
| 600 | 1 | 0 |
| 700 | 1 | 2 |
| 710 | 1 | 0 |
| 850 | 1 | 0 |
| 1000 | 2 | 1 |
| 1100 | 1* | 0 |
| 1150 | 2 | 3 |
| 1300 | 1* | 4 |
| 1400 | 1* | 0 |
| 2000 | 1 | 2 |
| 49.97km | 122 | 34 |

##### **S2 Table: The number of spotlighting surveys conducted in Tallaganda National Park between October and December 2021, the transect length and the number of individual southern greater gliders detected. Surveys were conducted on foot along tracks and roads. Number of observers differed for each survey.**

| Transect Length (m) | Number of Surveys | Number of Individuals |
| --- | --- | --- |
| 200 | 2 | 5 |
| 220 | 1 | 14 |
| 250 | 1 | 2 |
| 300 | 3 | 10 |
| 350 | 1 | 9 |
| 400 | 2 | 5 |
| 500 | 9 | 38 |
| 550 | 1 | 1 |
| 7.87km | 20 | 84 |

##### **S3 Table: List of arboreal mammal species that inhabit Tallaganda and East Gippsland, their scientific name, threatened status, and which study area their range overlaps with (EG = East Gippsland, T = Tallaganda). Threatened status is from the Commonwealth Environment Protection and Biodiversity Conservation Act 1999 (100).**

| Common Name | Scientific Name | Threatened Status | Study Area |
| --- | --- | --- | --- |
| Antechinus | *Antechinus* spp.  (Multiple species) | Not Listed | Both |
| Common Brushtail Possum | *Trichosurus vulpecula* | Not Listed | Both |
| Common Ringtail Possum | *Pseudocheirus peregrinus* | Not Listed | Both |
| Eastern Pygmy Possum | *Cercartetus nanus* | Not Listed | T |
| Feathertail Glider | *Acrobates* spp.  (Broad-toed or Narrow-toed) | Not Listed | Both |
| Mountain brushtail possum | *Trichosorus cunninghami* | Not Listed | Both |
| Southern Greater Glider | *Petauroides volans* | Endangered | Both |
| Inland Sugar Glider | *Petaurus notatus* | Not Listed | Both |
| Yellow-bellied glider | *Petaurus australis* | Vulnerable | EG |

##### **S4 Table: East Gippsland spotlighting transects, treatment (nestbox or control), direction of transect (PE = perpendicular to road or PA = parallel to road), the burn percentage (%), canopy burn (field measure; yes - canopy at the site was burnt, no – no canopy scorch visible), severity class according to the satellite fire mapping (96), land tenure, and Ecological Vegetation Class (Only the majority class is recorded here). To determine the fire severity class from the fire mapping, each transect was given a 50m buffer, and the class that covered the majority of the area was selected. * Not used in analysis. Site 11NB and Site 11C were removed entirely. Sites 16 to 20 were within the prescribed burn areas.**

| Site | Treatment | Direction of transect | Burn (%) | Canopy burn | Severity class | Tenure | Ecological Vegetation Class |
| --- | --- | --- | --- | --- | --- | --- | --- |
| 01C | Control | PE | 50 | Yes | High | Flora & Fauna Reserve | Shrubby Dry Forest |
| 01NB | Nestbox | PE | 80 | Yes | High | Flora & Fauna Reserve | Montane Dry Woodland |
| 02C | Control | PE | 100 | Yes | High | Flora & Fauna Reserve | Shrubby Dry Forest |
| 02NB | Nestbox | PE | 100 | Yes | High | Flora & Fauna Reserve | Shrubby Dry Forest |
| 03C | Control | PE | 100 | Yes | High | State Forest | Damp Forest |
| 03NB | Nestbox | PA | 100 | Yes | High | State Forest | Damp Forest |
| 04C | Control | PE | 100 | Yes | High | State Forest | Montane Dry Woodland |
| 04NB | Nestbox | PE | 100 | Yes | High | State Forest | Montane Dry Woodland |
| 05C | Control | PE | 100 | Yes | High | State Forest | Shrubby Dry Forest |
| 05NB | Nestbox | PE | 80 | Yes | High | State Forest | Shrubby Dry Forest |
| 06C | Control | PE | 95 | Yes | High | State Forest | Wet Forest |
| 06NB | Nestbox | PE | 80 | Yes | High | State Forest | Damp Forest |
| 07C | Control | PE | 15 | Yes | Low | National Park | Wet Forest |
| 07NB | Nestbox | PE | 10 | Yes | Low | National Park | Damp Forest |
| 08C | Control | PE | 100 | Yes | High | National Park | Damp Forest |
| 08NB | Nestbox | PE | 95 | Yes | High | National Park | Damp Forest |
| 09C | Control | PA | 20 | No | No Data | State Forest | Wet Forest |
| 09NB | Nestbox | PE | 20 | No | No Data | National Park | Wet Forest |
| 10C | Control | PA | 20 | No | Low | State Forest | Damp Forest |
| 10NB | Nestbox | PA | 30 | No | Low | State Forest | Damp Forest |
| 12C* | Control | PA | 70 | Yes | Low/No Data | National Park | Shrubby Dry Forest |
| 12NB* | Nestbox | PA | 100 | Yes | Low | National Park | Shrubby Dry Forest |
| 13C | Control | PE | 40 | No | Low | National Park | Damp Forest |
| 13NB | Nestbox | PE | 40 | No | Low | National Park | Wet Forest |
| 14C | Control | PE | 40 | No | Low | National Park | Wet Forest |
| 14NB | Nestbox | PE | 30 | No | Low | National Park | Wet Forest |
| 15C | Control | PE | 20 | No | Low | National Park | Wet Forest |
| 15NB | Nestbox | PE | 35 | No | Low | National Park | Wet Forest |
| 16C | Control | PA | 10 | No | No Data | State Forest | Montane Grassy Woodland |
| 16NB | Nestbox | PA | 20 | No | No Data | State Forest | Montane Grassy Woodland |
| 17C | Control | PE | 50 | No | No Data | State Forest | Montane Grassy Woodland |
| 17NB | Nestbox | PE | 50 | No | No Data | State Forest | Montane Dry Woodland |
| 18C | Control | PA | 20 | No | No Data | State Forest | Montane Dry Woodland |
| 18NB | Nestbox | PE | 10 | No | No Data | State Forest | Montane Dry Woodland |
| 19C | Control | PE | 30 | Yes | No Data | State Forest | Tableland Damp Forest |
| 19NB | Nestbox | PA | 10 | No | No Data | State Forest | Tableland Damp Forest |
| 20C | Control | PE | 50 | No | No Data | State Forest | Montane Dry Woodland |
| 20NB | Nestbox | PE | 80 | Yes | No Data | State Forest | Montane Dry Woodland |

##### **S5 Table: Tallaganda spotlighting transects, treatment (nestbox or control), the burn percentage (%), canopy burn (field measure; yes - canopy at the site was burnt, no – no canopy scorch visible), severity class according to fire mapping (58), land tenure, and vegetation class (Only the majority class is recorded here). To determine the fire severity class from the fire mapping, each transect was given a 50m buffer, and the class that covered the majority of the area was selected.**

| Site | Treatment |  | Burn (%) | Canopy Burn | Severity Class | Tenure | Vegetation Class |
| --- | --- | --- | --- | --- | --- | --- | --- |
| 01A | Nestbox |  | 15 | No | Low | National Park | Tableland and escarpment Moist herb/fern grass forest |
| 01B | Nestbox |  | 30 | No | Moderate | National Park | Tableland and escarpment Moist herb/fern grass forest |
| 01C | Control |  | 20 | Yes | Low | National Park | Tableland and escarpment Moist herb/fern grass forest |
| 02A | Nestbox |  | 15 | No | Moderate | National Park | Eastern Tablelands Acacia/Herb/Grass Forest |
| 02B | Nestbox |  | 30 | No | High | State Forest | Eastern Tablelands Acacia/Herb/Grass Forest |
| 02C | Control |  | 20 | No | High | National Park | Eastern Tablelands Acacia/Herb/Grass Forest |
| 03A | Nestbox |  | 30 | No | Low | National Park | Eastern Tableland Fern/Herb/Grass Moist Forest |
| 03B | Nestbox |  | 55 | No | Moderate | National Park | Eastern Tableland Fern/Herb/Grass Moist Forest |
| 03C | Control |  | 50 | Yes | Extreme | National Park | Eastern Tableland Fern/Herb/Grass Moist Forest |
| 04A | Nestbox |  | 100 | Yes | High | National Park | Eastern Tableland Fern/Herb/Grass Moist Forest |
| 04B | Nestbox |  | 90 | Yes | Extreme | National Park | Eastern Tableland Fern/Herb/Grass Moist Forest |
| 04C | Control |  | 100 | Yes | High | National Park | Eastern Tableland Fern/Herb/Grass Moist Forest |
| 05A | Nestbox |  | 50 | Yes | Moderate | National Park | Eastern Tableland Fern/Herb/Grass Moist Forest |
| 05B | Nestbox |  | 50 | Yes | Moderate | National Park | Eastern Tableland Fern/Herb/Grass Moist Forest |
| 05C | Control |  | 40 | Yes | Moderate | National Park | Eastern Tableland Fern/Herb/Grass Moist Forest |
| 06A | Nestbox |  | 60 | Yes | High | National Park | Eastern Tablelands Acacia/Herb/Grass Forest |
| 06B | Nestbox |  | 60 | Yes | High | National Park | Tableland and escarpment Moist herb/fern grass forest |
| 06C | Control |  | 70 | Yes | Moderate | National Park | Tableland and escarpment Moist herb/fern grass forest |
| 07A | Nestbox |  | 15 | No | Moderate | National Park | Eastern Tableland and Escarpment Shrub/Fern Dry Forest |
| 07B | Nestbox |  | 15 | No | Low | National Park | Eastern Tableland and Escarpment Shrub/Fern Dry Forest |
| 07C | Control |  | 15 | Yes | High | National Park | Tableland and escarpment Moist herb/fern grass forest |
| 08A | Nestbox |  | 50 | Yes | Moderate | National Park | Eastern Tableland Fern/Herb/Grass Moist Forest |
| 08B | Nestbox |  | 50 | Yes | Extreme | National Park | Tableland and escarpment Moist herb/fern grass forest |
| 08C | Control |  | 40 | Yes | High | National Park | Eastern Tablelands Acacia/Herb/Grass Forest |
| 09A | Nestbox |  | 90 | Yes | High | National Park | Tableland and escarpment Moist herb/fern grass forest |
| 09B | Nestbox |  | 100 | Yes | Extreme | National Park | Eastern Tableland and Escarpment Shrub/Fern Dry Forest |
| 09C | Control |  | 80 | Yes | High | State Forest | Eastern Tableland Fern/Herb/Grass Moist Forest |
| 10A | Nestbox |  | 30 | No | Moderate | National Park | Eastern Tablelands Dry Shrub Forest |
| 10B | Nestbox |  | 50 | Yes | High | National Park | Tableland and escarpment Moist herb/fern grass forest |
| 10C | Control |  | 70 | Yes | High | National Park | Eastern Tablelands Dry Shrub Forest |

##### **S1 Fig: The design and layout of each study site, (1NB) nestbox site and (1C) control site. Both nestbox and control sites were marked out with flagging and reflective tape in the same way.**


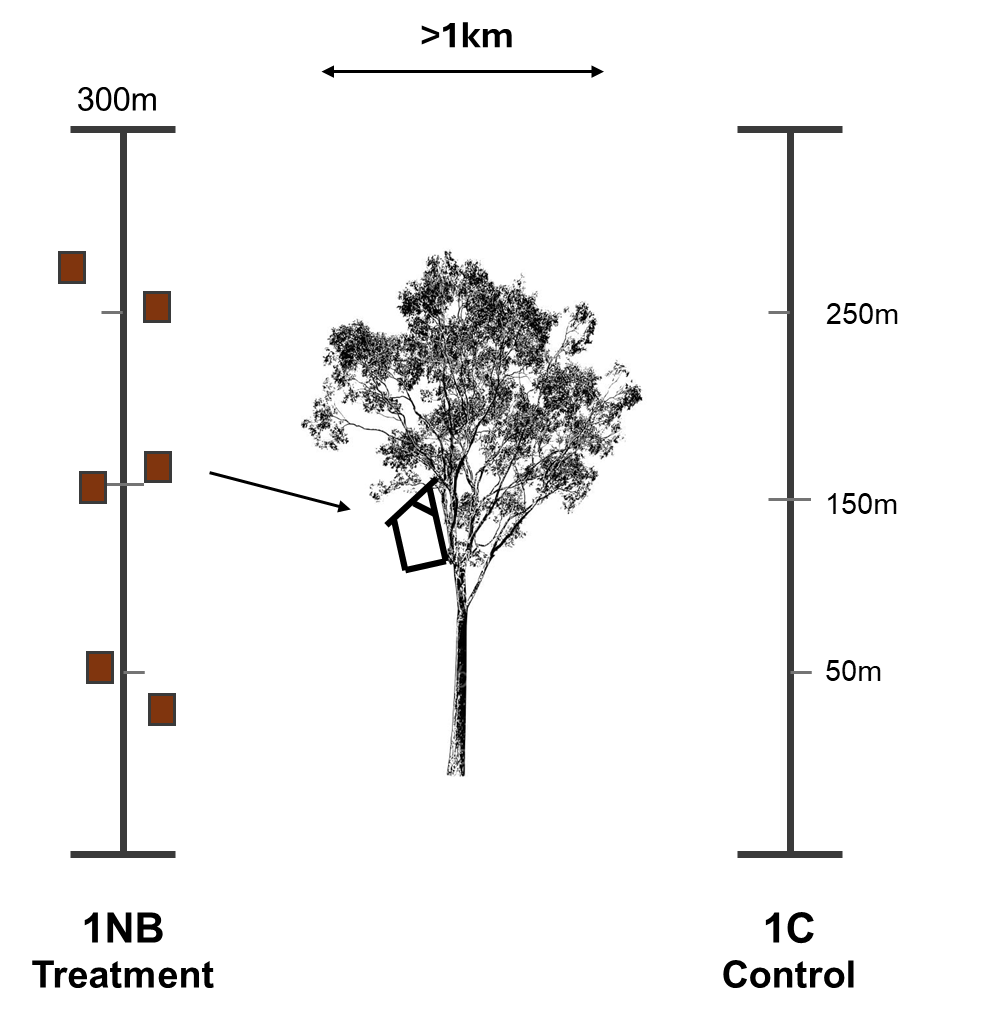


##### **S6 Table: The percentage of sites where each species was detected using combined data from two rounds of spotlighting surveys and camera trapping at nestbox sites. There are 26 cameras, 13 sites used in Tallaganda and 28 cameras, 14 sites used for East Gippsland.**

|  | East Gippsland | | Tallaganda | |
| --- | --- | --- | --- | --- |
|  | Spotlighting | Camera Monitoring | Spotlighting | Camera Monitoring |
| Antechinus | 0 | 35.71 | 0 | 7.7 |
| Bat | 0 | 42.86 | 0 | 0 |
| Common Brushtail Possum | 41.67 | 0 | 36.67 | 7.7 |
| Common Ringtail Possum | 8.33 | 0.00 | 2.00 | 0 |
| Feathertail Glider | 8.33 | 100 | 10.00 | 53.85 |
| Inland Sugar Glider | 8.33 | 85.71 | 3.33 | 23.08 |
| Mountain Brushtail Possum | 33.33 | 42.86 | 6.67 | 0 |
| Southern Greater Glider | 41.67 | 57.14 | 96.67 | 69.23 |
| Yellow-bellied Glider | 2.78 | 21.43 | 0 | 0 |

**
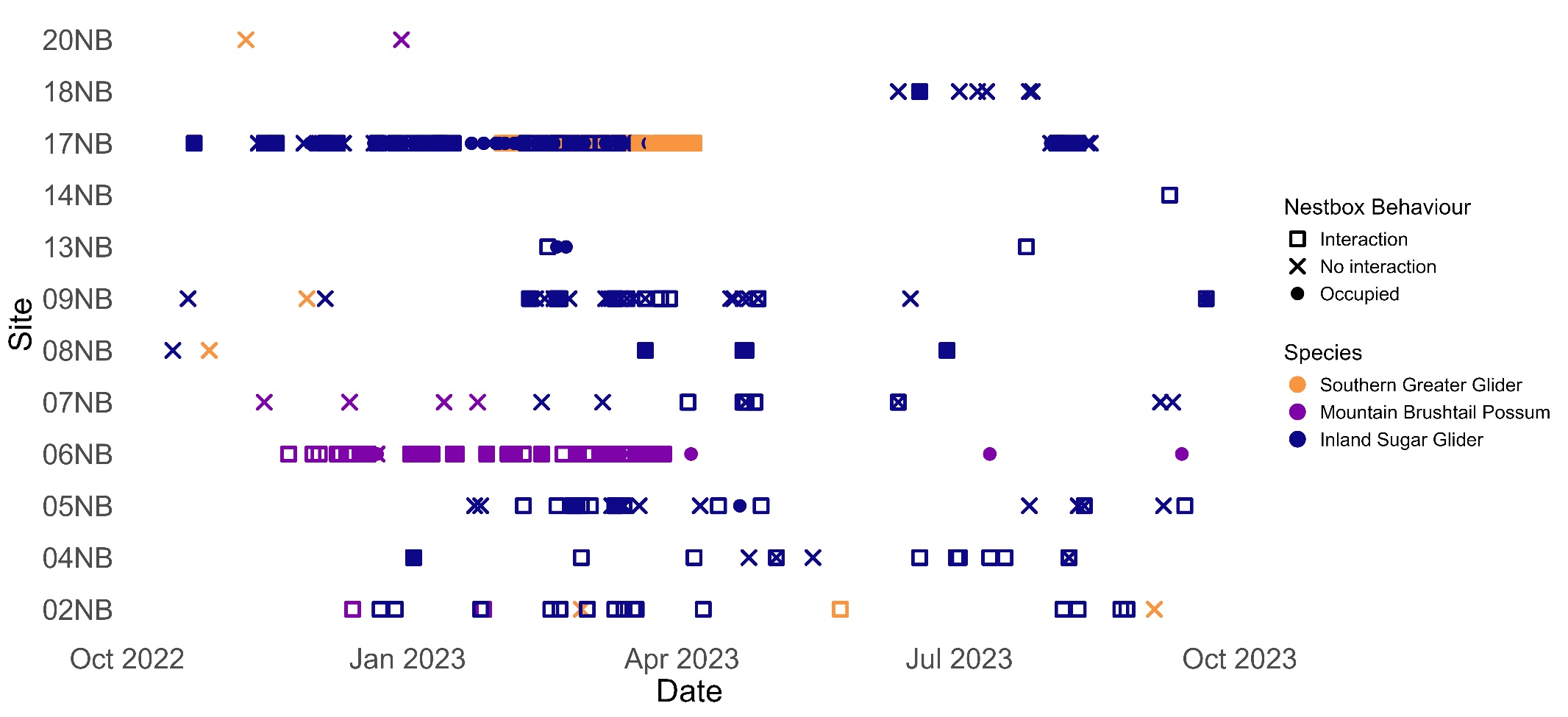
S2 Fig: Camera detections of southern greater gliders (orange), inland sugar gliders (blue) and mountain brushtail possums (purple) at nestbox sites over time in East Gippsland, Victoria. Each site displays the combined species detections from the two nestbox cameras per site. Animal behaviour at the nestbox is split into no interaction (cross), interaction (square) or use (filled circle). Feathertail gliders were excluded from this figure for clarity.**

##### **S3 Fig: Camera detections of southern greater gliders (orange), inland sugar gliders (blue) and feathertail gliders (pink) at nestbox sites over time in Tallaganda, New South Wales. Each site displays the combined species detections from the two nestbox cameras per site. Animal behaviour at the nestbox is split into no interaction (cross), interaction (square) or use (filled circle).**


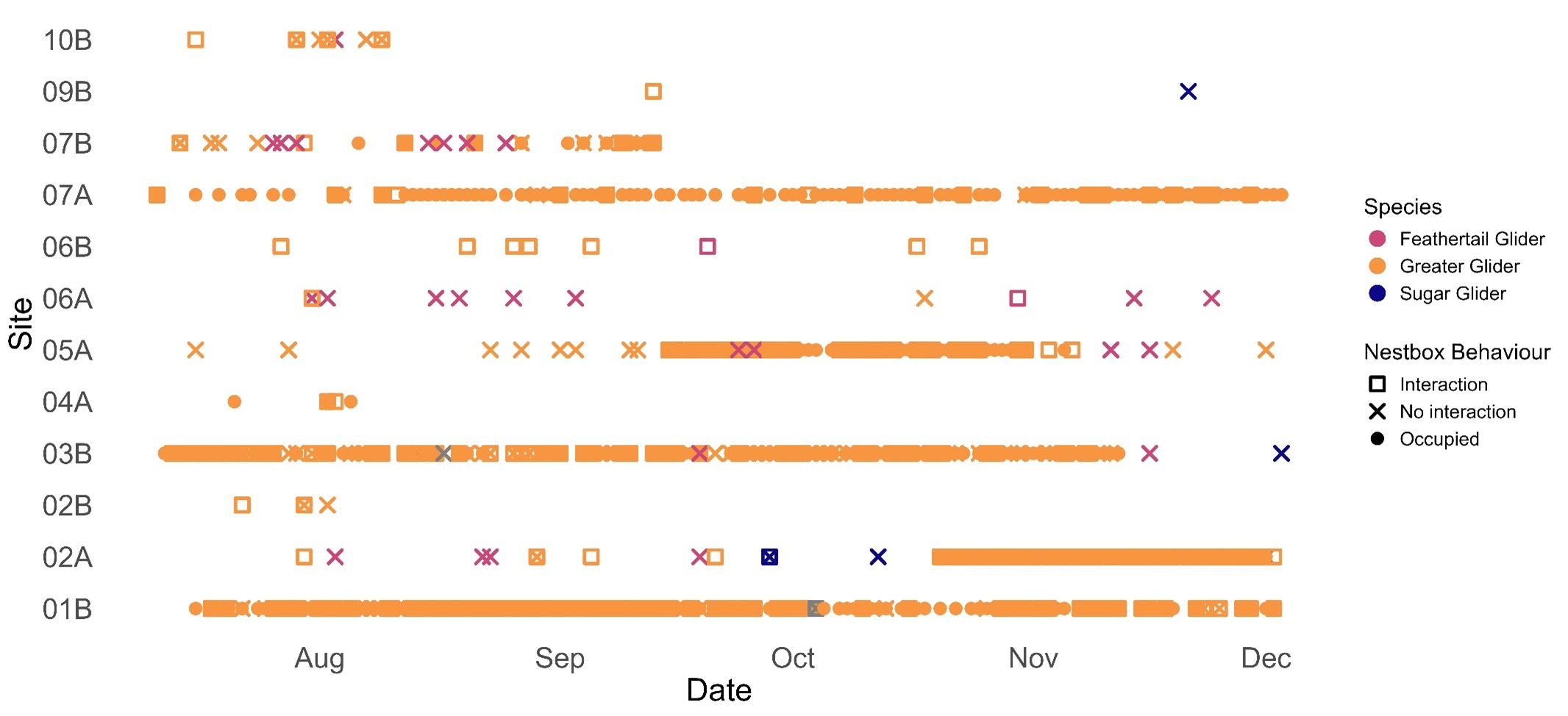

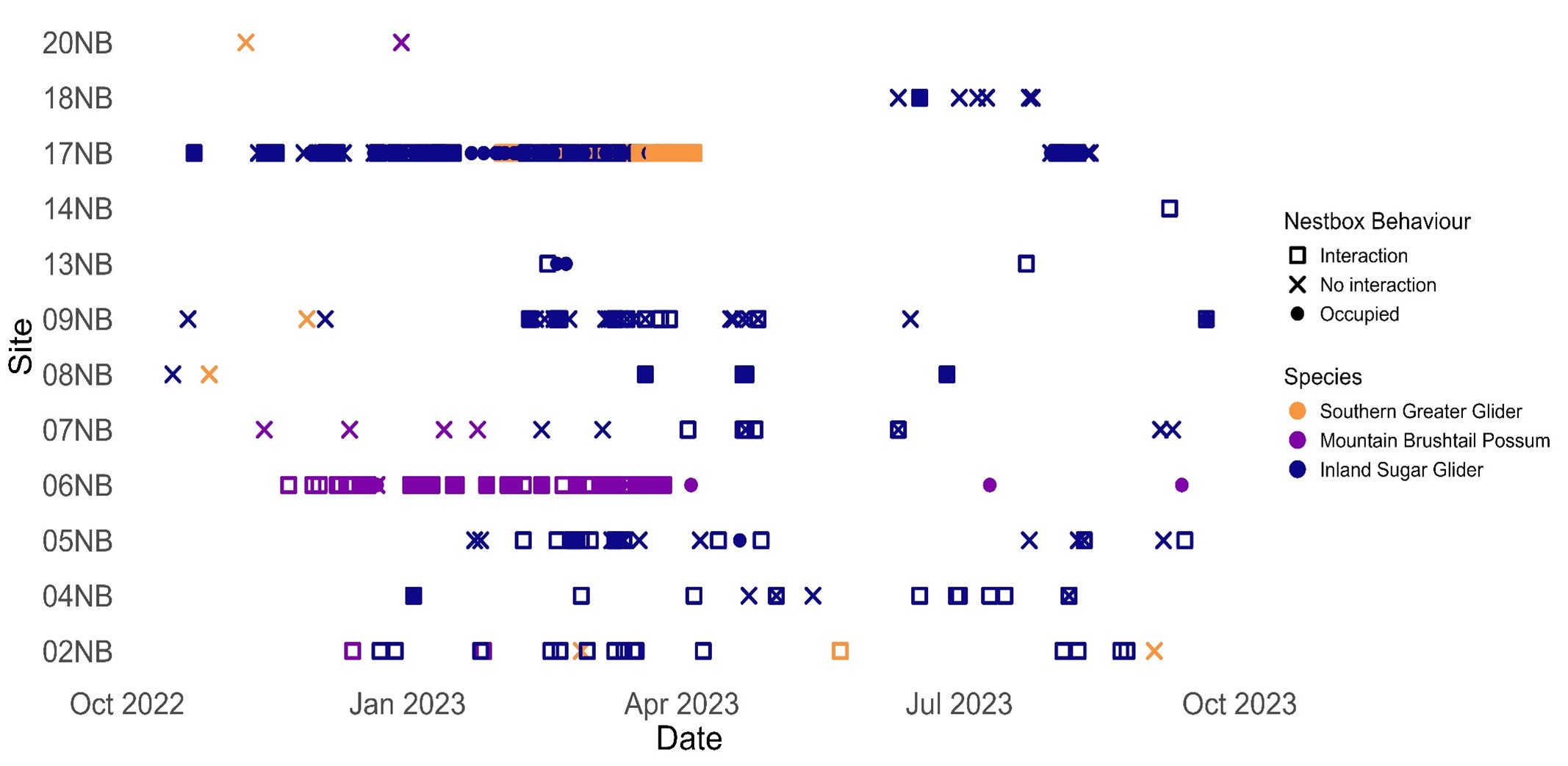


##### **S4 Fig: The predicted mean abundance of southern greater gliders at nestbox and control sites before (pre) and after (post) nestbox deployment. Predicted means and standard error bars are shown for each study site, generated from the generalised linear mixed model results presented in Table 2.**


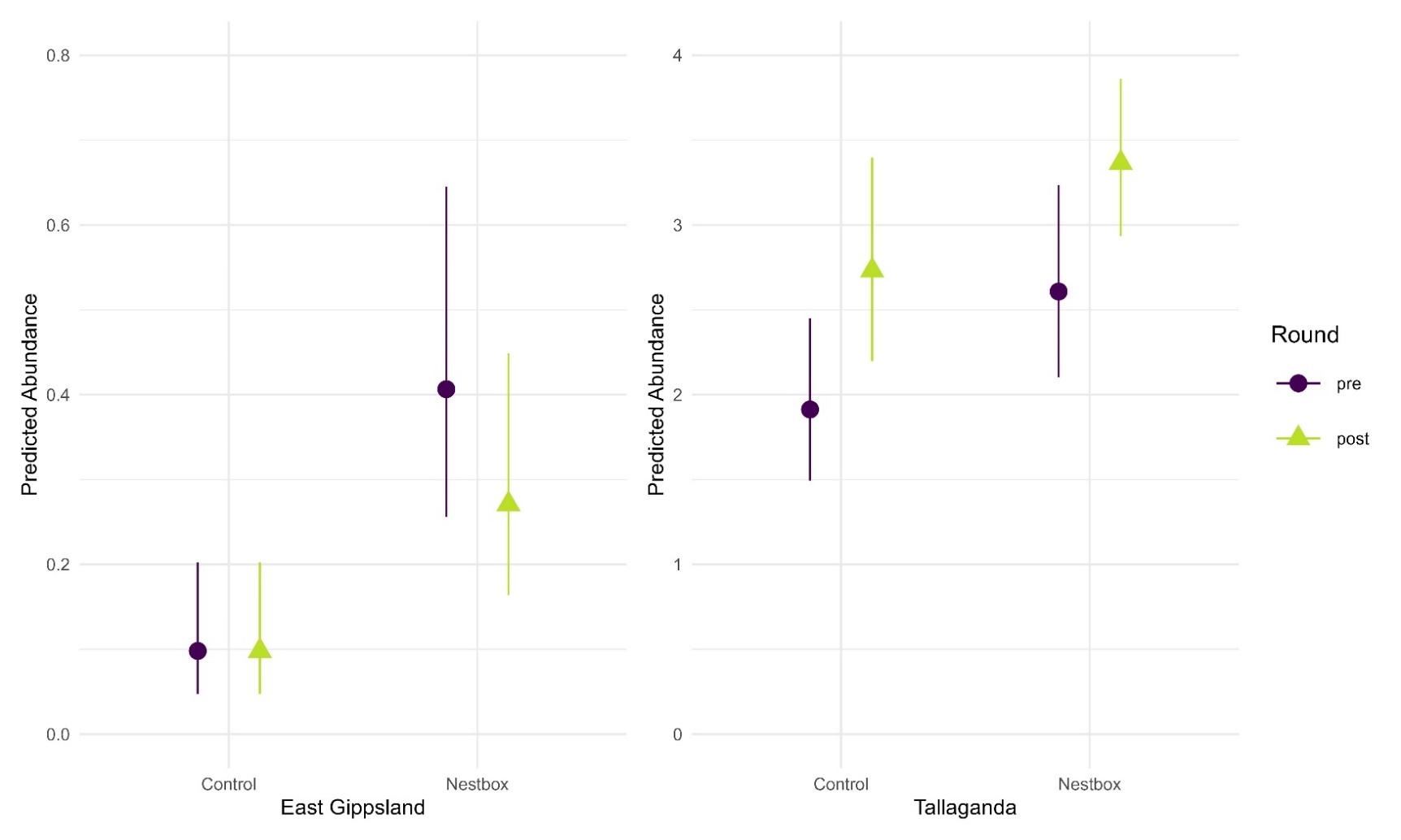


##### 
